# CoPi/Co(OH)_2_ Modified Ta_3_N_5_ as New Photocatalyst for Photoelectrochemical Cathodic Protection of 304 Stainless Steel

**DOI:** 10.3390/ma12010134

**Published:** 2019-01-03

**Authors:** Xuan Xie, Li Liu, Emeka E. Oguzie, Ying Li, Fuhui Wang

**Affiliations:** 1 Shenyang National Laboratory for Materials Science, Northeastern University, NO. 3-11, Wenhua Road, Heping District, Shenyang 110819, Liaoning, China; xxie14s@imr.ac.cn (X.X.); fhwang@imr.ac.cn (F.W.); 2 Institute of Metal Research, Chinese Academy of Sciences, 62# Wencui Road, Shenyang 110016, Liaoning, China; liying@imr.ac.cn; 3 Electrochemistry and Materials Science Research Laboratory, Department of Chemistry, Federal University of Technology Owerri, Owerri PMB 1526, Nigeria; oguziemeka@yahoo.com

**Keywords:** Ta_3_N_5_ nanorod-arrays, CoPi/Co(OH)_2_ modification, cathodic protection, photocatalysis

## Abstract

In this work, CoPi and Co(OH)_2_ nanoparticles were deposited on the surface of Ta_3_N_5_ nanorod-arrays to yield a novel broad-spectrum response photocatalytic material for 304 stainless steel photocatalytic cathodic protection. The Ta_3_N_5_ nanorod-arrays were prepared by vapor-phase hydrothermal (VPH) and nitriding processes and characterized by scanning electron microscopy (SEM), X-ray diffraction (XRD), X-ray photoelectron spectroscopy (XPS), and UV-Vis spectroscopy, respectively, to obtain morphologies, crystal structures, surface compositions, and light response range. In order to analyze the performance improvement mechanism of CoPi/Co(OH)_2_ on Ta_3_N_5_ nanorod-arrays, the electrochemical behavior of modified and unmodified Ta_3_N_5_ was obtained by measuring the open circuit potential and photocurrent in 3.5 wt% NaCl solution. The results revealed that the modified Ta_3_N_5_ material better protects 304 stainless steel at protection potentials reaching −0.45 V.

## 1. Introduction

Since Fujishima and Honda [1] first reported the photoelectric effect of TiO_2_ in 1972, photocatalytic materials have become increasingly important, with diverse applications in several industrial processes [2,3,4,5,6]. Research has shown that many common photocatalytic materials, such as TiO_2_ [5,7], ZnO [8,9], and SnO_2_ [10], have wide band gaps, which means that they have almost only ultraviolet light response and have a low utilization rate of sunlight in practical applications. What’s more, it is difficult for a single photocatalyst to function effectively independently, even for rather simple processes like decomposition of water. In the same vein, single photocatalyst materials do not function efficiently in electrochemical protection applications. Photocatalytic activity can often be improved by regulating crystal surface [11] and surface defects [12], as well as by noble metal deposition [13,14] or semiconductor combination [15,16], which often result in photocatalytic composite systems with improved performance. Another approach involves exploration, design, and development of novel photocatalytic materials.

Some recent studies have focused on developing a new class of photocatalytic materials that respond readily to a broad range of spectra and can make full use of visible light to achieve photocatalysis. A material like BiVO_4_, for instance, with a band gap of 2.4 eV, has a large visible region response and successfully degrades organics in visible light [17].

Ta_3_N_5_ is a novel photocatalytic material with a broad spectral response range [18,19,20,21]. Ta_3_N_5_, prepared by Hara et al. [22], could oxidize water into O_2_ efficiently, with maximum quantum yields of 10%. Luo et al. [23] reported that in the visible region nano Au/Ta_3_N_5_ composite showed a significantly enhanced photocatalytic activity for hydrogen evolution from water. Zhen et al. [24] reported that a Ta_3_N_5_ nanorod modified with Co(OH)_x_ had a strong absorption in the visible light range up to 620 nm and could achieve high photon-to-current conversion efficiency. The use of catalyst promoters like Co(OH)_x_ [25,26] and CoPi [27,28] has become an important and promising way to improve the photocatalytic capability of photocatalysts [29,30,31,32,33]. Such promoters function by restraining the recombination rate of interfacial photogenerated electron-hole pairs and play a useful role in providing ready sites for the oxidation half reaction [34].

In the 1990s, Tsujikawa and Yuan [5] first proposed a TiO_2_ photocatalytic coating technique for cathodic corrosion protection of carbon steel. Since then, the use of photocatalysts in cathodic protection has continued to attract attention within the scientific community and different photocatalytic materials have been investigated for metal corrosion protection efficacy. Sun et al. [35] reported that a C_3_N_4_-In_2_O_3_ nanocomposite with quasi-shell-core structure provides photoelectrochemical cathodic protection for the coupled 304 stainless steel (SS) under visible light. Wang et al. [36] observed that Bi_2_Se_3_/TiO_2_ nanocomposites successfully exhibited great photogenerated cathodic protection performance for 304 stainless steel.

However, we have not seen any reports on the use of Ta_3_N_5_ materials for photoelectrochemical cathodic protection of metals. Compared to the previous work on Ta_3_N_5_, it is necessary and meaningful to explore the cathodic protection performance of this material with a broad spectral response range.

In this work, a vapor-phase hydrothermal process and subsequent nitriding treatment were used to prepare Ta_3_N_5_ nanorod-array films, which were subsequently modified by addition of CoPi/Co(OH)_2_ and subjected to microstructure characterization. The change of photoelectrochemical cathodic protection properties of Ta_3_N_5_ material before and after modification were compared using electrochemical methods. This study provides a theoretical basis for the application of novel photocatalytic materials with broad spectral response ranges in cathodic protection.

## 2. Experimental Section

### 2.1. Specimen Preparation and Modification

Vapor-phase hydrothermal (VPH) is a process to grow metal oxides on the corresponding metal matrix, which has become a very attractive method in recent years [37,38]. It has been used in growth of ZnO nanotube and nanorod-array films on a zinc foil substrate [37] and growth of rutile nanorod and titanate nanotubes on a titanium foil substrate [38].

In this work, based on previous methods, one-dimensional Ta_2_O_5_ nanorod-arrays were synthesized on a tantalum foil substrate (10 mm × 15 mm) by the VPH process, and these arrays were subsequently converted to Ta_3_N_5_ nanorod-array films by NH_3_ nitriding treatment [24].

To obtain the Ta_3_N_5_ material modified by CoPi/Co(OH)_2_, double co-catalysts, CoPi and Co(OH)_2_, were deposited as follows: CoPi was loaded on the surface of Ta_3_N_5_ nanorod-array films by photoelectric chemical deposition [28], and then Co(OH)_2_ was modified by chemical deposition modification on top of CoPi [24].

### 2.2. Characterization of Ta_3_N_5_

The surface morphologies of the Ta_3_N_5_ nanorod-array films were observed and analyzed using an INSPECT F50 (FEI Co., Hillsboro, OR, USA) field emission scanning electron microscope (SEM), while the growth thickness of the films was also observed by cross-section morphologies at a working voltage of 25 kV. The crystalline structures were measured through a X’pert PRO (Panalytical, Almelo, The Netherlands) X-ray diffractometer (XRD) using Cu Kα radiation at 40 kV, with 2θ ranging from 10° to 90°. In order to identify if CoPi/Co(OH)_2_ was successfully loaded, the changes of surface elemental compositions were analyzed by ESCALAB250 (Thermo VG, Waltham, MA, USA) X-ray photoelectron spectroscopy (XPS) with Al Kα radiation. The ultraviolet-visible spectra of the Ta_3_N_5_ films were collected by a diffuse reflectance V-770 (JASCO, Tokyo, Japan) UV-Vis spectrophotometer in the wavelength range 200–900 nm.

### 2.3. Preparation of Ta_3_N_5_ Electrode

The Ta_3_N_5_ nanorod-array films were fabricated into electrodes before use in photoelectrochemical performance tests for metal protection. The Ta_3_N_5_ electrode and 304 stainless steel electrode were cut in the same size (10 mm × 15 mm). One side of each sample was exposed to the corrosive solution and the other sides covered with a resin-paraffin mixture (1:1). The samples were pasted and fixed to copper wires as electrodes. A 3.5 wt% NaCl solution was used as electrolyte solution in order to simulate the seawater environment.

### 2.4. Characterization of Photoelectrochemical Performance

**Figure 1:**
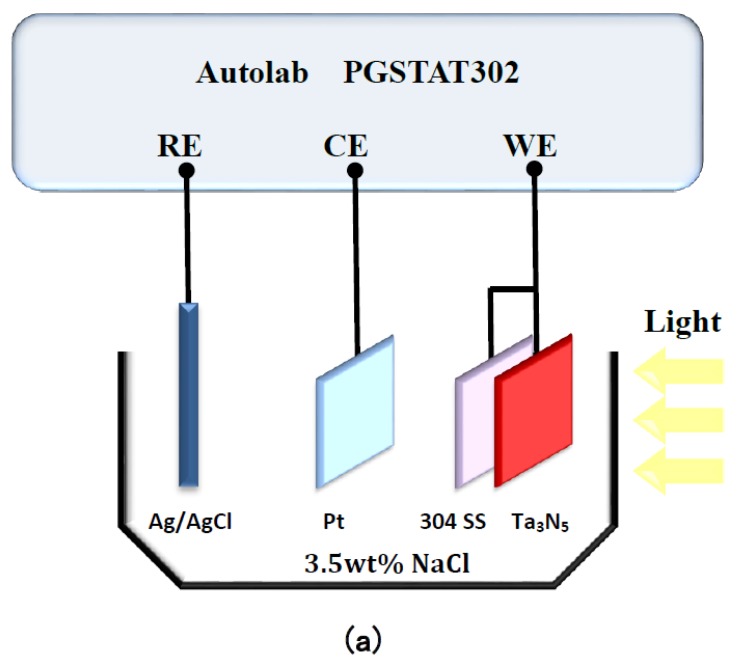

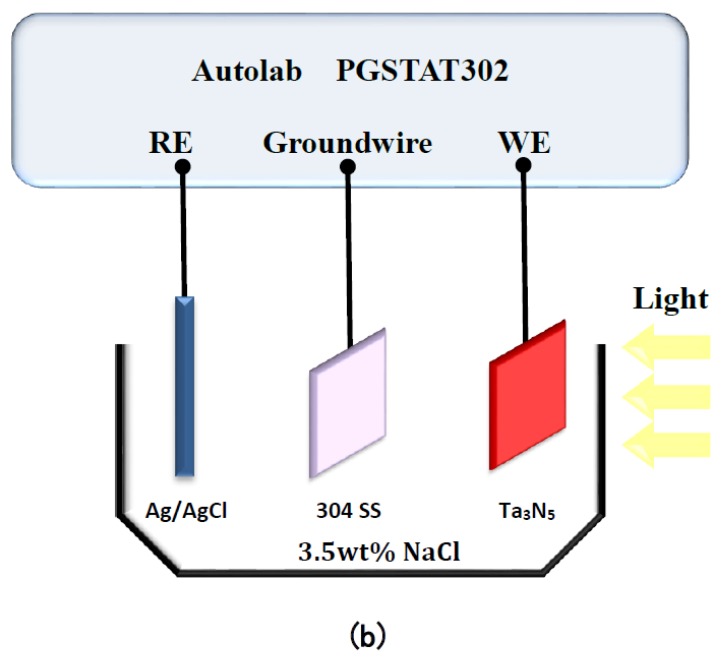
Schematic diagram of the Autolab electrochemical test system for measuring (**a**) open circuit potentials and (**b**) photocurrent densities of Ta_3_N_5_ films.

Characterization of photoelectrochemical performance was conducted using a PGSTAT302N potentiostat Autolab (Metrohm Autolab, Utrecht, The Netherlands). As shown in Figure 1a, a Pt electrode and a KCl-saturated silver/silver chloride electrode (Ag/AgCl) were connected as counter electrode and reference electrode, respectively. The Ta_3_N_5_ electrodes with and without CoPi/Co(OH)_2_ modification were coupled with 304 stainless steel and used as working electrodes. This connection method enabled detection of the open circuit potential (OCP) changes over time in the dark and under illumination, respectively.

The electrochemical noise module (ECN) was used to measure the photogenerated current of the two kinds of Ta_3_N_5_ electrodes as a function of time, in the dark and under illumination. As shown in Figure 1b, the working electrode Ta_3_N_5_ was placed opposite the 304 stainless steel counter electrode (connected to the ground wire in ECN measurements, so that electrons can flow from the Ta_3_N_5_ electrode to the 304 stainless steel electrode); the Ag/AgCl electrode was connected likewise as reference electrode. All the measured potentials were relative to the Ag/AgCl electrode (0.1981 V). The electrolyte, 3.5 wt% NaCl solution, was replaced for each new measurement. The light source was a 300 W PLS-SXE 300 Xe lamp (Beijing PerfectLight Co. Ltd., Beijing, China). The experimental temperature was at room temperature.

## 3. Results and Discussion

### 3.1. Micromorphologies of Ta_3_N_5_

**Figure 2:**
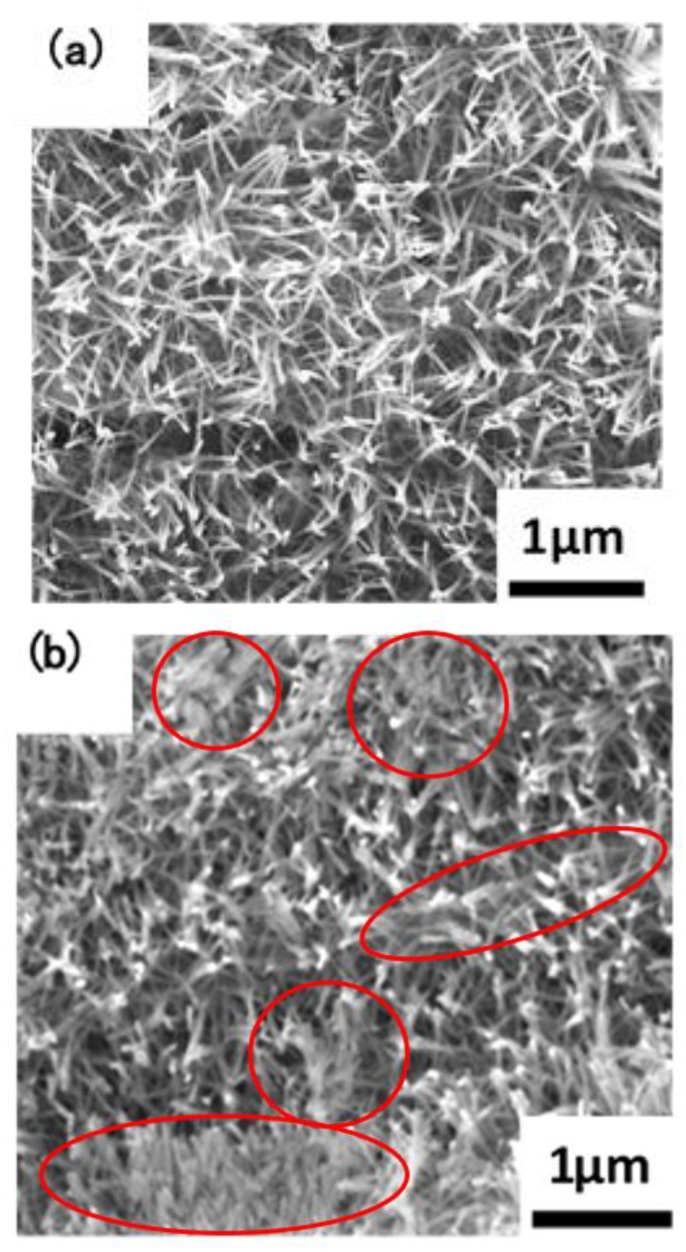

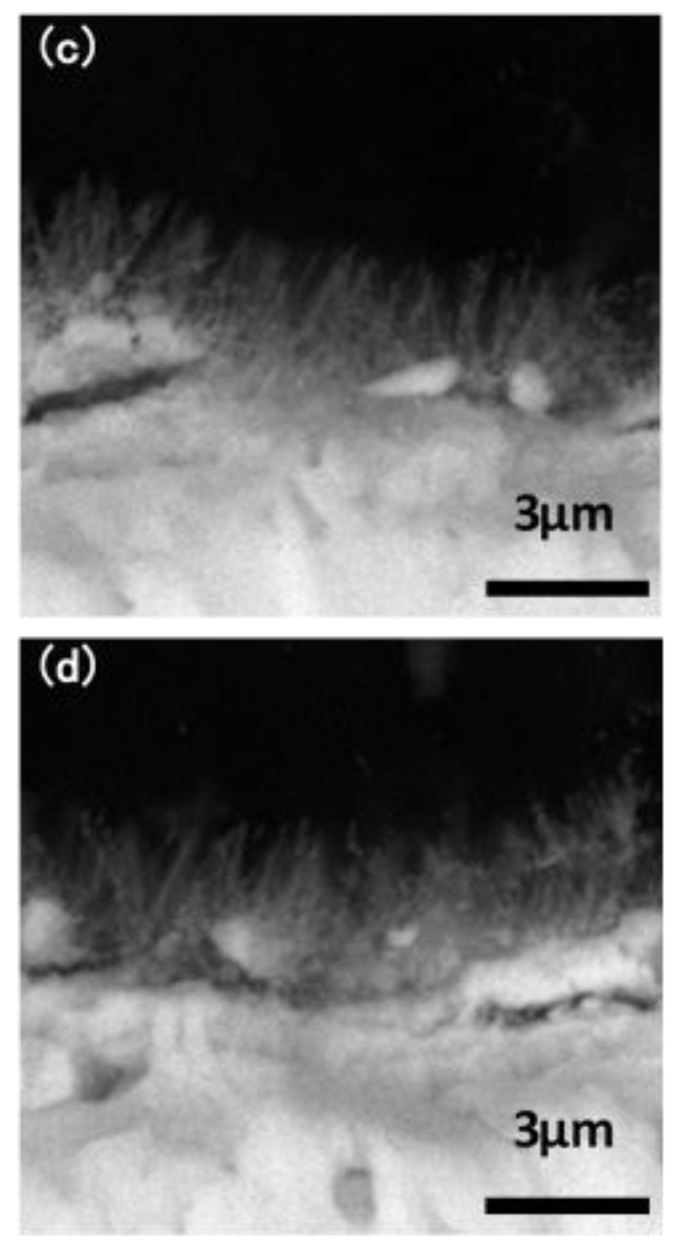
SEM images of the microstructures of the prepared Ta_3_N_5_ nanorod-array films: (**a**) surface morphology of unmodified Ta_3_N_5_; (**b**) surface morphology of modified Ta_3_N_5_; (**c**) cross-section morphology of unmodified Ta_3_N_5_; (**d**) cross-section morphology of modified Ta_3_N_5_.

The macroscopic surface of the Ta_3_N_5_ material prepared by the VPH method and nitriding treatment was a uniform and wine-red film. The micromorphologies of the unmodified Ta_3_N_5_ (shown in Figure 2) grew in the form of a vimineous nanorod-array, continuously and closely connected to each other (Figure 2a). The corresponding cross-section morphology shows the nanorod-arrays to be almost vertical, with lengths of approximately 2–3 µm (Figure 2c).

After surface modification with CoPi/Co(OH)_2_, the surface morphology shows evidence of agglomeration of some nanorods (Figure 2b), suggesting that CoPi/Co(OH)_2_ probably adsorbed on nanorod-arrays and caused them to bind together as shown in the marked areas. The corresponding cross-section morphology in Figure 2d shows similar features as the unmodified Ta_3_N_5_ nanorod-arrays in Figure 2c, and the nanorod agglomeration is not very clear.

Such nanorod-array structure should impart many merits like excellent conductivity and prominent quantum size effect, high specific surface area, and a large number of surface reaction sites, which will promote transport of electro-hole pairs. This means that solar energy conversion efficiency and light absorption ability will be enhanced, with the possibility of improved electrochemical protective performance.

### 3.2. Crystal Structure and Chemical Composition Analysis of Ta_3_N_5_

**Figure 3:**
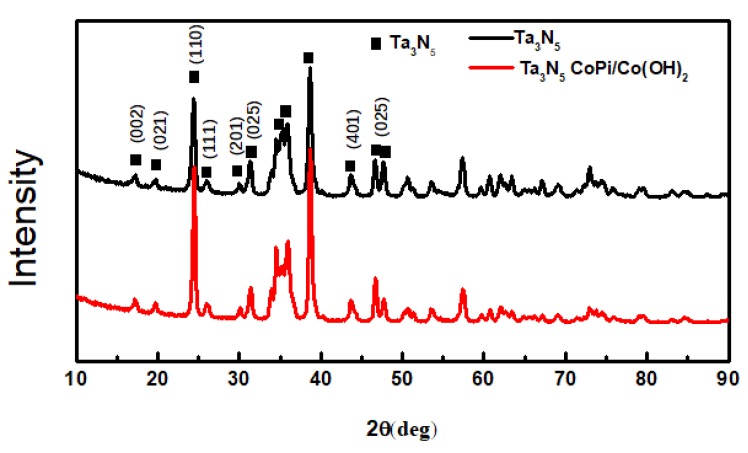
X-ray diffraction patterns of the Ta_3_N_5_ material with and without CoPi/Co(OH)_2_ modification.

The XRD patterns of the as-prepared Ta_3_N_5_ material with and without CoPi/Co(OH)_2_ modification are shown in Figure 3. When 2θ is at 17.21°, 19.74°, 24.47°, 26.00°, 30.09°, 33.85°, 34.49°, 36.01°, 38.68°, 43.67°, 46.67°, and 47.75°, the diffraction peaks are basically consistent with the standard Tantalum Nitride Ta_3_N_5_ (JCPDS card no. 19-1291). These peaks match with the (002), (021), (110), (111), (201), (025,) (401), and (025) crystal planes completely. The other weaker peaks include the Ta matrix and a small amount of tantalum nitride compounds of other valences arising from the nitriding treatment. A comparison of the modified and unmodified Ta_3_N_5_ materials reveals that the diffraction peak position and peak intensity of the two curves almost coincide. Moreover, no peaks are observed for CoPi/Co(OH)_2_, indicating that their amounts may be too low to be detected by XRD. This could also imply that the CoPi/Co(OH)_2_ additives merely adhered to surface of the Ta_3_N_5_ nanorod-array, causing the observed agglomeration.

**Figure 4:**
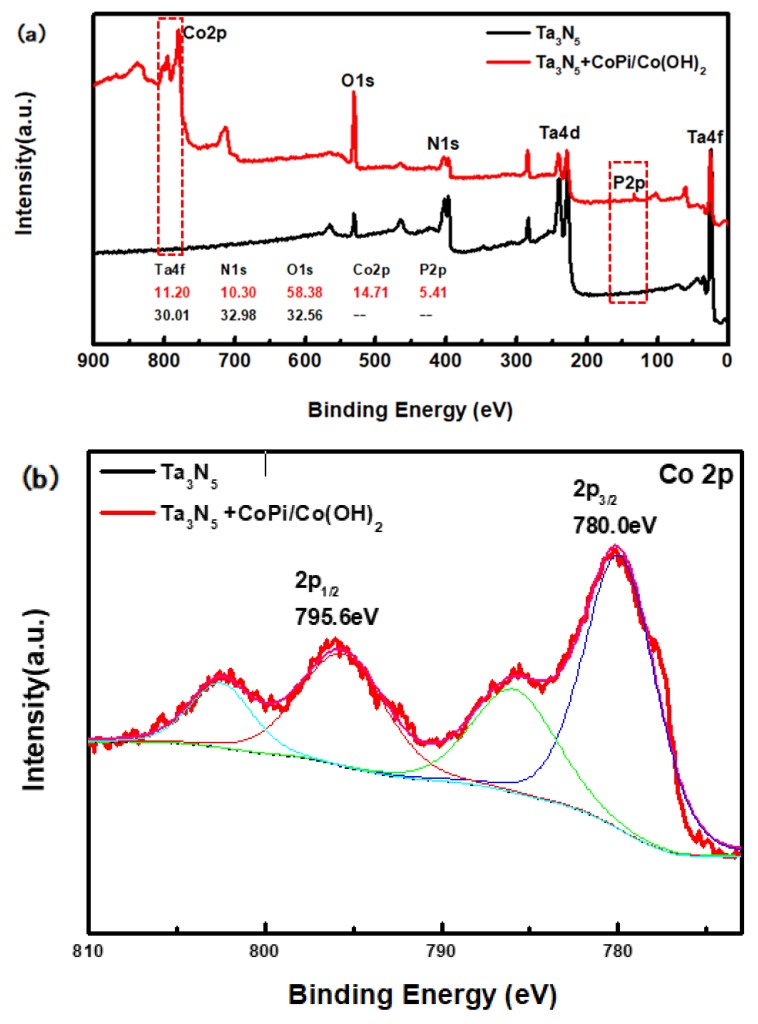

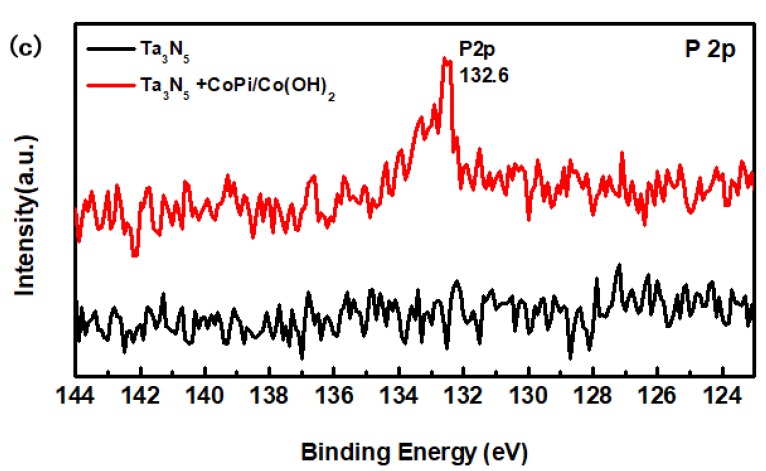
X-ray photoelectron spectroscopy (XPS) spectra of the Ta_3_N_5_ material with and without CoPi/Co(OH)_2_ modification: (**a**) survey spectrum, (**b**) Co 2p peak, (**c**) P 2p peak.

The compositions of the modified and unmodified Ta_3_N_5_ surfaces were further investigated by XPS, with special focus on the peaks and transformations of Co and P elements. The high-resolution complete survey XPS in Figure 4a shows Ta 4f, Ta 4d, N 1s, O 1s, Co 2p, and P 2p peaks, corresponding to the as-prepared samples. The major difference between the two curves is that the peak of the Co and P appeared only in the curve of the modified Ta_3_N_5_, indicating that CoPi/Co(OH)_2_ was indeed successfully deposited on the surface of the Ta_3_N_5_ nanorod-array in the modified sample.

From the high-resolution spectra of CoPi/Co(OH)_2_ for the modified Ta_3_N_5_ (Figure 4b), the binding energies of Co 2p3/2 and 2p1/2 (the red curve) are 780.0 eV and 795.6 eV, respectively. Furthermore, the peak of P 2p at 132.6 eV could be mainly attributed to the P element in CoPi in Figure 4c (the red curve). Both sets of results confirm the presence of CoPi/Co(OH)_2_ on the modified Ta_3_N_5_.

### 3.3. UV-Vis Absorption Properties of Ta_3_N_5_

**Figure 5:**
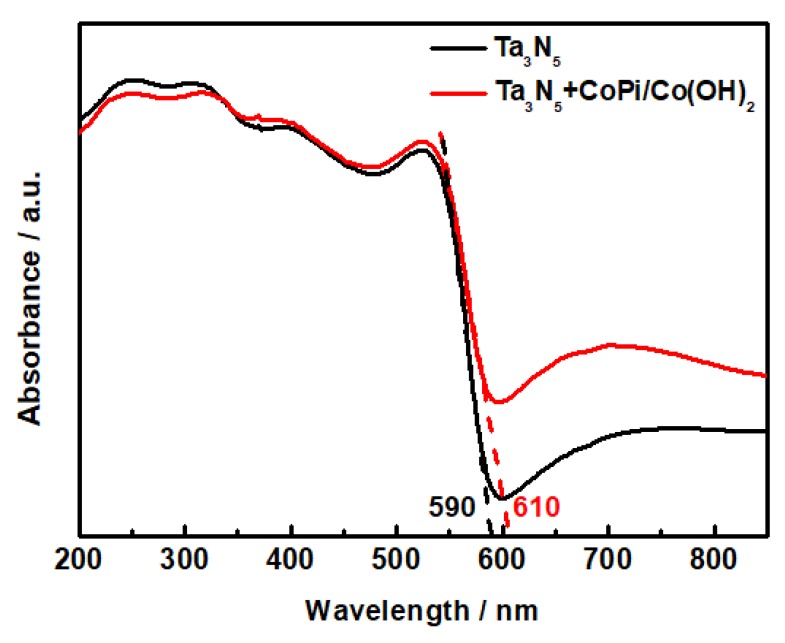
UV-Vis absorption spectra of the Ta_3_N_5_ material with and without CoPi/Co(OH)_2_ modification.

The UV-Vis diffuse reflectance spectrum of the Ta_3_N_5_ nanorod-array films with and without modification is shown in Figure 5. The absorption shoulders of both Ta_3_N_5_ curves are located deep into the visible region. The absorption spectra of the unmodified and CoPi/Co(OH)_2_ modified Ta_3_N_5_ are 590 nm and 610 nm, respectively, corresponding to band gap energies Δ*Eg* = 2.10 eV and 2.03 eV according to the equation $Eg=\frac{1240}{\lambda }$. The light absorption threshold of the CoPi/Co(OH)_2_ surface-modified Ta_3_N_5_ is wider than the unmodified one, hence the narrowed band gap would result in lower photogenerated electron transition energy, stronger photocatalytic activity, and better photoelectric chemical protection performance in theory.

Ta_3_N_5_ is a new type of photocatalytic material with a broad spectral response range, which has a much higher visible absorption value and narrower energy gap than that of the TiO_2_ photocatalyst. Hara et al. [22] reported the photocatalytic mechanism and energy band structure of TaON, Ta_2_O_5_, and Ta_3_N_5_ materials and ascribed the small energy gaps of TaON and Ta_3_N_5_ to the higher potential energy of the N 2p orbitals compared to the O 2p orbitals, resulting in the higher negative potential of Ta_3_N_5_ and the narrowing of the semiconductor energy gap.

### 3.4. Photoelectrochemical Cathodic Protection Performance of Ta_3_N_5_

**Figure 6:**
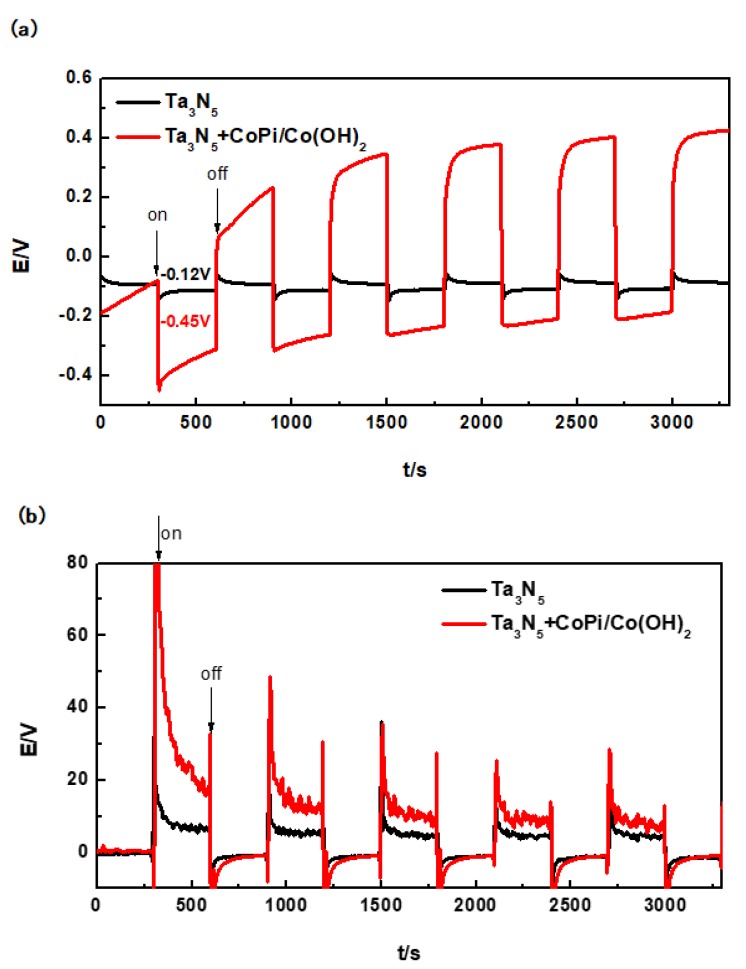
Evolution of open circuit potentials (**a**) and photocurrent densities (**b**) of modified and unmodified Ta_3_N_5_ material with and without illumination.

After coupling of the Ta_3_N_5_ electrode (with or without CoPi/Co(OH)_2_ modification) and the 304 stainless steel electrode, their open circuit potentials over time in 3.5 wt% NaCl solution in the dark and under the illumination were tested, as shown in Figure 6a. The duration of either cycle (in the dark and under the illumination) was 300 s, which means that a complete cycle lasted for 600 s.

The corrosion potential of the 304 stainless steel was initially determined to be approximately −0.17 V in 3.5 wt% NaCl solution by potentiodynamic polarization measurements. The unmodified Ta_3_N_5_ also has photocatalytic activity [23,24,39] and theoretically should generate some degree of protective potential. However, the actual results in Figure 6a show that the potential of the unmodified Ta_3_N_5_ could only reach to −0.12 V after the first illumination (300 s). It remained stable under illumination and did not change with the continued illumination. This means that the OCP of the unmodified Ta_3_N_5_ (−0.12 V) is more positive than that of 304 stainless steel (−0.17 V). Therefore, the unmodified Ta_3_N_5_ cannot achieve electrochemical protection of 304 stainless steel.

For the Ta_3_N_5_ modified with CoPi/Co(OH)_2_, the first photoinduced potential drop reached −0.45 V after 300 s, which is far lower (more cathodic) than the corrosion potential of 304 stainless steel, indicating that the generation and transfer of photogenerated electrons occurred instantaneously. Under this condition, there are no photogenerated holes to start reacting with OH^−^ and H_2_O in solution, and the electrochemical protection of 304 stainless steel should be largely realizable. Although this potential increases slowly to −0.20 V with prolonged illumination for 3000 s (at the end of illumination cycle), it still remained more negative than the corrosion potential of 304 stainless steel. Our results therefore prove that Ta_3_N_5_ modified with CoPi/Co(OH)_2_ achieved photoelectrochemical cathodic protection of 304 stainless steel within the experimental time interval. However, the rising trend of its potential also indicates that CoPi/Co(OH)_2_ is probably not stable and its ability to consume holes decreases on the surface of Ta_3_N_5_ nanorods. Ta_3_N_5_ material is also unstable and easy to be oxidized by photogenerated holes due to the accumulation of holes, so that its photoelectrochemical protective performance is still unstable.

The minimum protection current density of 304 stainless steel is 15 µA/cm^2^ [29]. As shown in Figure 6b, the instantaneous photocurrent of unmodified Ta_3_N_5_ can reach to 32 µA/cm^2^ (300 s), but it remains stable at about 7 µA/cm^2^ (600 s). This illustrates that the stable current cannot reach to 15 µA/cm^2^, so that the 304 stainless steel cannot be protected, consistent with the change law of OCP.

There is an instantaneous rise to more than 80 µA/cm^2^ that rapidly drops to 15 µA/cm^2^ within one illumination cycle, and is just sufficient to protect 304 stainless steel within the first cycle. For subsequent cycles, the photocurrent density at the beginning of illumination consistently remained above 20 µA/cm^2^, and then dropped to around 10 µA/cm^2^ at the end of cycle. It is therefore obvious that CoPi/Co(OH)_2_ modification improved the photocurrent of Ta_3_N_5_ consistent with the OCP results, and can thus be further investigated as a method for improving the performance of photocatalytic materials. Although the CoPi/Co(OH)_2_ modified Ta_3_N_5_ does protect 304 stainless steel, the long-term performance would require further improvement.

### 3.5. Mechanism Analysis of Photoelectrochemical Cathodic Protection

**Figure 7:**
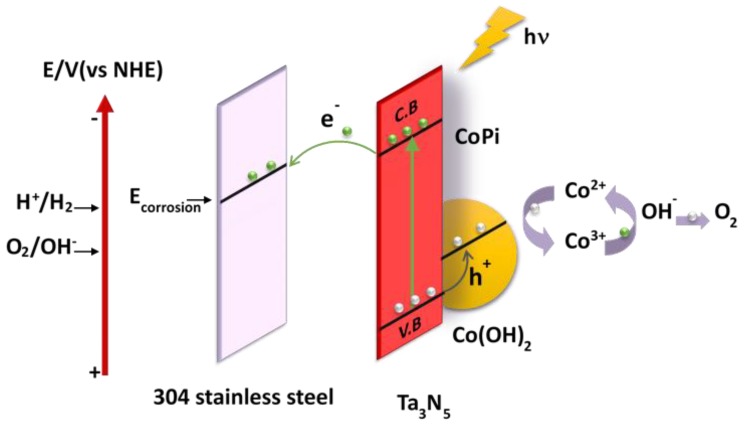
Proposed mechanism of photoelectrochemical cathodic protection of 304 stainless steel by CoPi/Co(OH)_2_ modified Ta_3_N_5_ films under illumination.

The proposed mechanism of photoelectrochemical cathodic protection for 304 stainless steel under illumination by Ta_3_N_5_ modified with CoPi/Co(OH)_2_ is schematically illustrated in Figure 7. Photogenerated electrons from the Ta_3_N_5_ valence band (VB) are excited to the conduction band (CB) under illumination, leaving photogenerated holes and electrons in the valence band and the conduction band, respectively. Subsequently, the photogenerated electrons transfer to the surface of the 304 stainless steel matrix to reduce oxygen, causing the surface potential of the 304 stainless steel to fall below its corrosion potential, thus realizing photoelectrochemical cathodic protection. At the same time, the photogenerated holes transfer from the VB to the CoPi/Co(OH)_2_ additive to oxidize Co^2+^ into Co^3+^ [28].

CoPi is strongly adsorbed on the surface of Ta_3_N_5_ and provides more adsorption sites and reaction sites in the reaction process. Co(OH)_2_ is oxidized from Co^2+^ into Co^3+^, and then the Co^3+^ oxidizes H_2_O to O_2_. During this process, Co^3+^ is reduced to Co^2+^, which again awaits to be reoxidized by the next photogenerated hole. This cycle does not include Co^2+^ consumption. The valence transitions between Co^2+^ and Co^3+^ of the CoPi/Co(OH)_2_ co-catalysis is favorable for separation and migration of photogenerated charges [33,40,41], and as a result, more holes are consumed, photogenerated charge recombination and the reverse reaction is inhibited, and electron-hole pairs are increased [34,42,43]. Finally, the photostability and photogenerated electrons and holes utilization efficiency of Ta_3_N_5_ material are enhanced so that the protective effect on metals is improved.

## 4. Conclusions

The photoelectrochemical cathodic protection of 304 stainless steel in a 3.5 wt% NaCl solution by a novel broad-spectrum response material—CoPi/Co(OH)_2_ modified Ta_3_N_5_—was studied. The main conclusions are as follows: CoPi/Co(OH)_2_ modified Ta_3_N_5_ can attain a more negative corrosion potential and the minimum protection photocurrent density of 304 stainless steel and as such can theoretically achieve photoelectrochemical cathodic protection for 304 stainless steel. CoPi/Co(OH)_2_, as co-catalyst, can reduce the activation energy of Ta_3_N_5_, promote photogenerated charge separation, consume holes and provide more active sites for electrochemical reactions, thus improving the photoelectric property and greatly enhancing the electrochemical protection for metals. However, long-term performance would require further improvement due to the decline of protection current density at the end of the later illumination cycles.
